# The likelihood ratio and its graphical representation

**DOI:** 10.11613/BM.2019.020101

**Published:** 2019-04-15

**Authors:** Farrokh Habibzadeh, Parham Habibzadeh

**Affiliations:** 1 Managing Director, R&D Headquarters, Petroleum Industry Health Organization, Shiraz, Iran; 2 Persian Bayangene Research and Training Center, Shiraz, Iran; 3 Student Research Committee, Shiraz University of Medical Sciences, Shiraz, Iran

**Keywords:** ROC curve, diagnostic tests, likelihood ratio

## Abstract

Diagnostic tests are important clinical tools. Bayes’ theorem and Bayesian approach are important methods for interpreting test results. The Bayesian factor, the so-called likelihood ratio, has not always been well-understood. In this article, we try to discuss the likelihood ratio and its value for a specific test result, a positive or negative test result, and a range of test results, along with their graphical representations.

## Introduction

First described in 1763, Bayes’ theorem, named after Reverend Thomas Bayes (an English statistician and philosopher), is now one of the cornerstones of methods used for interpreting diagnostic test results. In mathematical terminology, it is presented as follows in equation (Eq.) 1:
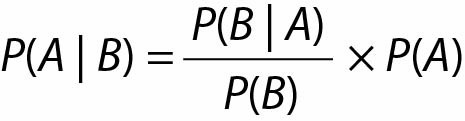
provided *P*(*B*) ≠ 0, and where *A* and *B* are two events, *P*(*A*) represents the probability that *A* happens, and *P*(*A | B*) is the conditional probability of *A* happens given the *B* has happened (*1*).

## Likelihood ratio

Suppose that *A* is the presence (*D*^+^) or absence (*D^–^*) of a disease and that *B* is the condition the result of a diagnostic test (*x*) fulfils, say the test result being equal to the value *r*. Based on Eq. 1, the probability of the presence of a disease (*D^+^*) given a test value *r* is:


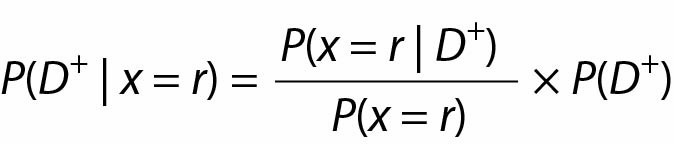


The probability of the absence of the disease (*D^–^*) given the test result equals to *r* is therefore:


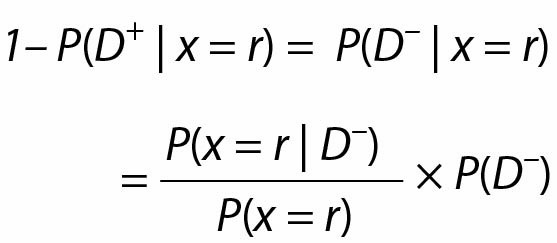


Dividing Eq. 2 by Eq. 3, and replacing *P*(*D^–^*) with 1 – *P*(*D^+^*) gives:
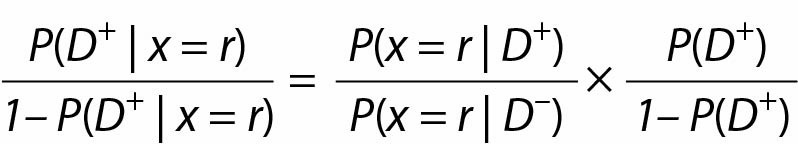
which is:


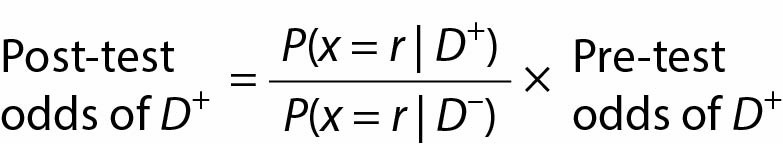


the well-known equation used in Bayesian approach to interpret test results (*2*). The factor *P*(*x = r | D^+^*) */ P*(*x = r | D^–^*) is termed the likelihood ratio (*LR*) when the test result equals to *r* and is represented as *LR*(*r*) (*1*). Generally speaking, the likelihood ratio indicates how many times more (or less) likely a certain condition for a test result is expected to be observed in diseased, compared with non-diseased, people (*3*). Four general possible conditions include likelihood ratio for a certain test value, likelihood ratio for a positive or negative test, and likelihood ratio for a range of test values (Table 1). To better understand the concept, let us examine the graphical representation of *LR*(*r*).

**Table 1:** Likelihood ratio for various test value conditions

**Likelihood ratio for**	**Notation**	**Definition**	**Graphical representation and equation**
**Certain test value of r**	*LR*(*r*)	The probability of observing a test value equal to *r* in diseased compared with non-diseased people	Slope of the tangent line to the ROC curve at the point corresponding to *r*; 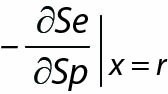
**Positive test (a test value equal to or more than a set cut-off value)**	*LR*(+)	The probability of observing a positive test in diseased compared with non-diseased people	Slope of the line segment joining the origin of the unit square to the point on the ROC curve corresponding to *r*; 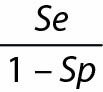
**Negative test (a test value less than a set cut-off value)**	*LR*(–)	The probability of observing a negative test in diseased compared with non-diseased people	Slope of the line segment joining the point on the ROC curve corresponding to *r* to the upper-right corner of the unit square 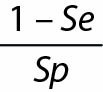
**A range of test values**	*LR*(Δ)	The probability of observing test values within a certain range in diseased compared with non-diseased people	Slope of the line segment joining the two points on the ROC curve corresponding to the upper and lower limits of the range 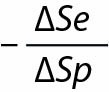
*Se* - sensitivity. *Sp* - specificity.			

## Graphical representation

### Likelihood ratio for a specific test result

**Figure 1:**
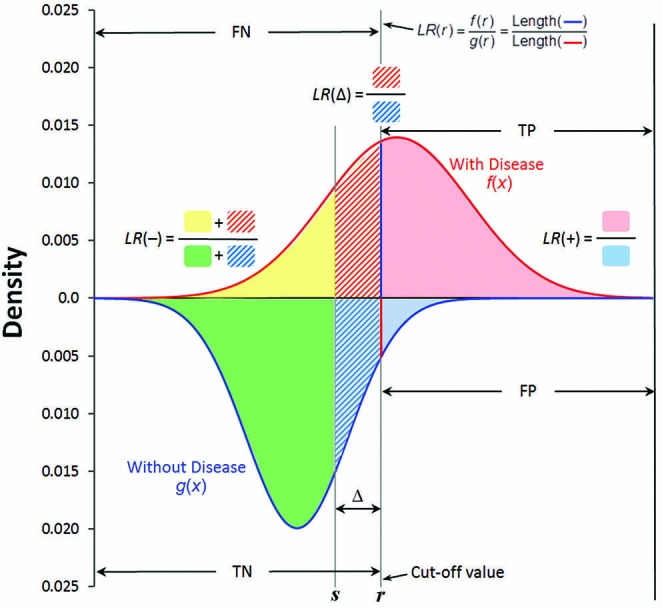
The probability density functions of a diagnostic test with continuous results for diseased, *f*(*x*), and non-diseased, *g*(*x*), persons. On the horizontal axis are test values with an arbitrary unit. Graphically, the likelihood ratio is generally a ratio of two areas, except for the *LR*(*r*), which is the ratio of two lengths. There are two test values, *r* and *s* (in our example FBS of 98 and 93 mg/dL, respectively, on the *x* axis). For the calculation of *LR*(+) and *LR*(–), *r* was considered the cut-off value. FN – false negative. TP – true positive. TN – true negative. FP – false positive.

Let *f*(*x*) and *g*(*x*) be the probability density function of a hypothetical diagnostic test with continuous results (*x*) for diseased (*D^+^*) and non-diseased (*D^–^*) population (Figure 1), respectively. We arbitrarily chose the test values having normal distribution for both the diseased and non-diseased population, although the functions can theoretically have any distributions. Each point of the test result (*x*) can be considered a cut-off value. Previously, we showed that the test sensitivity (*Se*) and specificity (*Sp*) are functions of the cut-off value as follows (*4*):


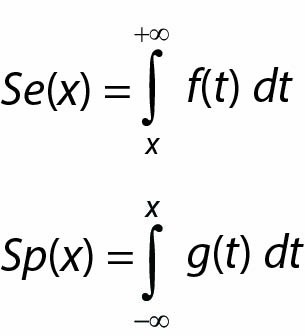


Assume that we set our cut-off value at *x* = *r*. *Se* is indeed the area under the curve *f*(*x*) to the right of the cut-off value *r* (the pink area in Figure 1). Then, by definition, partial derivative of the *Se* with respect to *x* is:


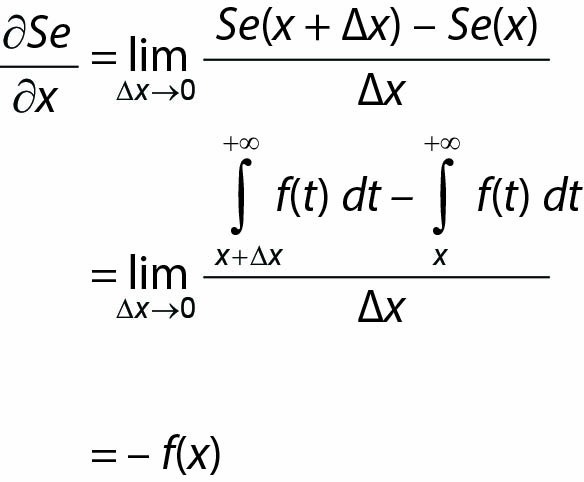


The minus sign before *f*(*x*) is because *Se* is a decreasing function of the cut-off value—*Se* decreases as cut-off value increases (*4*).

In a similar way, the partial derivative of *Sp* with respect to *x* can be derived:


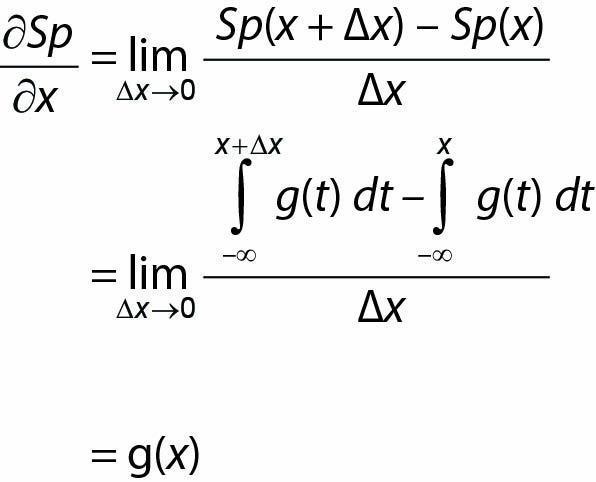


By definition:


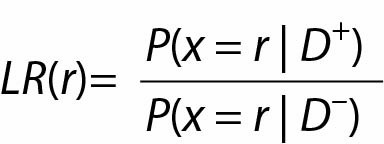


However, considering that *f*(*x*) and *g*(*x*) are density functions illustrating the distribution of the result values in the diseased and non-diseased population (Figure 1), we have:


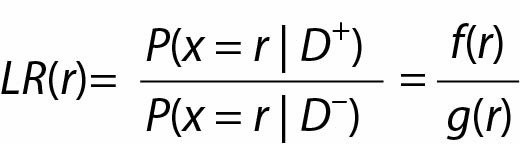


**Figure 2:**
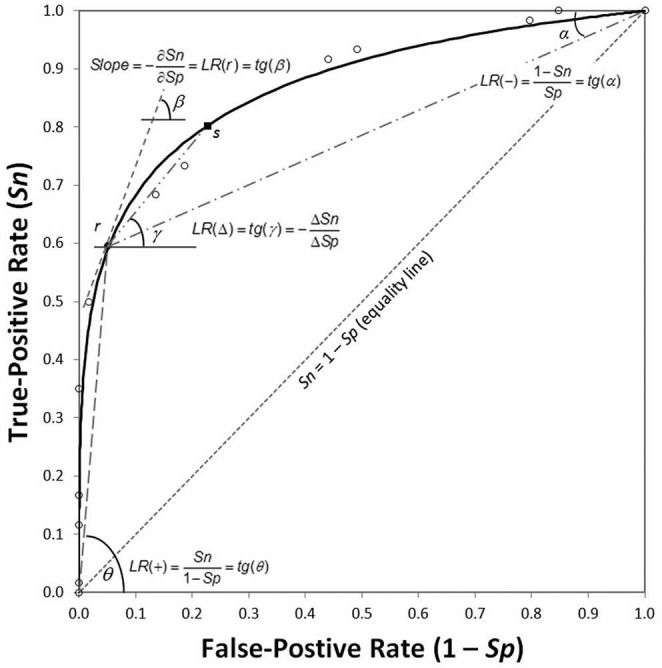
The ROC curve (solid black line) fitted to the data points (open circles) assuming the test value has a binormal distribution (Figure 1). The slope of the tangent line to the ROC (grey short dashed line) at the solid circle, the point corresponding to a test value *r* (FBS = 98 mg/dL in our example) in Figure 1, is the likelihood ratio of having an FBS of 98 mg/dL. Assuming a cut-off value of 98 mg/dL for FBS for the diagnosis of diabetes mellitus, the likelihood ratio of having a positive test, *LR*(+), is the slope of the line joining the origin to the solid circle (grey long dashed line). The likelihood ratio of a negative test, *LR*(–), is the slope of the line joining the solid circle to the upper-right corner (grey dash dotted line). The slope of the line segment joining the solid circle to the solid square (grey dash dot dotted line) is the likelihood ratio of having a test value between *s* and *r* (Figure 1). *Se* - sensitivity. *Sp* - specificity.

Before going further, there is a technical point worth to mention: from the theoretical point of view, the probability that a continuous random variable (here, *x*) will assume a particular value (here, *r*) is zero. Therefore, in the above equation, the statement *x = r* should be construed as *r – h* ≤ *x* ≤ *r + h*, when *h* approaches zero. Combining Equations 7 and 8, then:


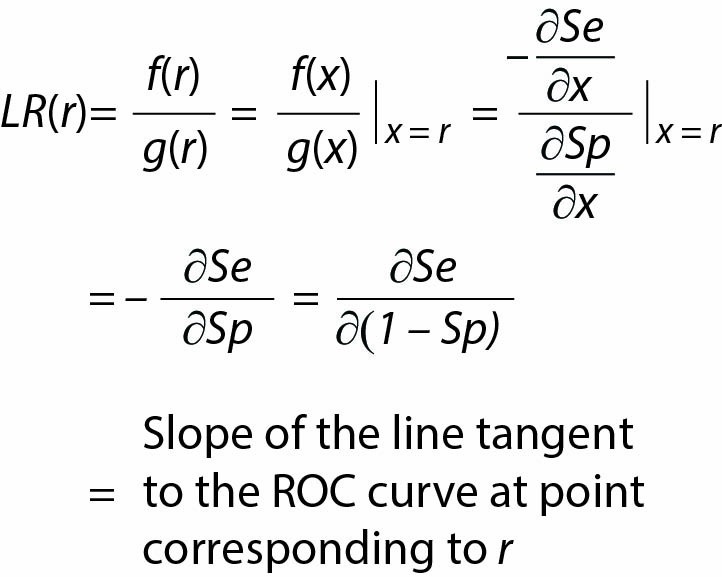


meaning that the likelihood ratio that the test result equals to the value *r*, *LR*(*r*), is equal to the slope of the line tangent to the receiver operating characteristic (ROC) curve (grey short dashed line, Figure 2) at the point corresponding to the test cut-off value, *r* (Table 1) (*4*).

Although *LR*(*r*) might provide useful information, its precise derivation is not generally possible in practice, unless a large database is available (*5*). The ROC curve is practically drawn from a set of discrete data that cannot be well fitted to a function; we just have a few discrete points. Although these points can be joined by various methods (line segments, spline, curve fitting, *etc*.), the curve is not differentiable and thus, in practice, it is not possible to determine the exact slope of the curve at a given point based on the available data (*4*-*6*). This makes accurate derivation of *LR*(*r*) very difficult, even impossible.

### Likelihood ratio for a positive/negative test result

Although determination of the likelihood ratio for a test value of *r* is difficult, we can easily derive the likelihood ratio for test values equal to or more than *r* or tests with dichotomous results—positive or negative. Suppose that the value *r* is the test cut-off value. This means test values equal to or more than *r* is considered positive (*T^+^*); otherwise the test result is considered negative (*T^–^*). The positive likelihood ratio, *LR*(+), is:


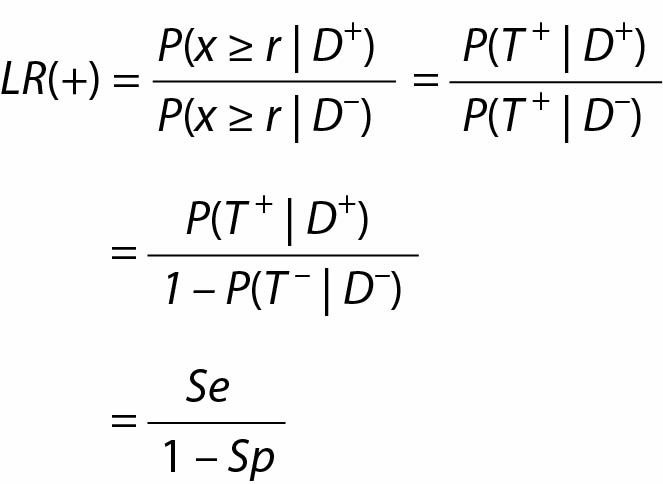


Graphically, *LR*(*+*) is the area under the curve *f*(*x*) to the right of the cut-off line (true-positive rate = *Se*) divided by the area under the curve *g*(*x*) to the right of the cut-off line (false-positive rate = 1 – *Sp*) (Figure 1). Mathematically, it is (*4*):


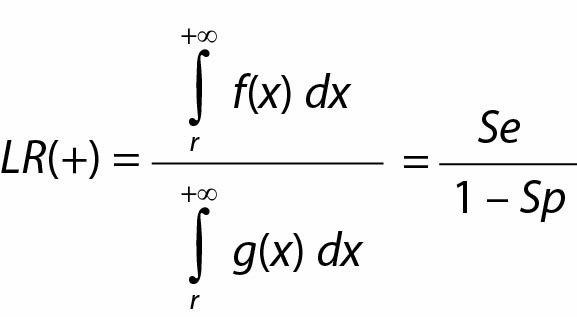


*LR*(*+*) is then clearly, the slope of the line segment joining the origin of the unit square to the point on the ROC curve corresponding to the test cut-off value, *r* (the solid circle, Figure 2, and Table 1).

There is a long-standing confusion between *LR*(*r*) and *LR*(*+*) in scientific literature. Some authors repeatedly have mentioned that *LR*(*+*) is equal to the slope of the cut-off point on the ROC curve, whereas, it is really the slope of the line joining the origin of the unit square to the cut-off point (Figure 2) (*7*-*11*). Although Choi has already addressed this misunderstanding, herein, we try to make things more clear, using a graphical approach, in hope to provide ways for better understanding the key concepts of the likelihood ratio (*5*).

In a similar way, the negative likelihood ratio, *LR*(*–*), can be calculated as:


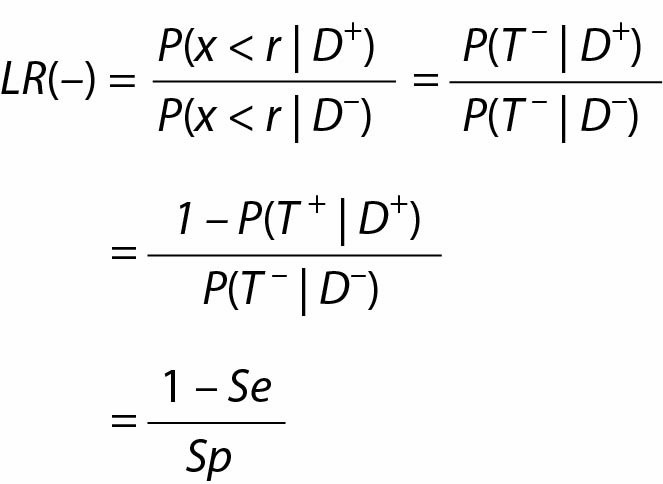


In other words (*4*),


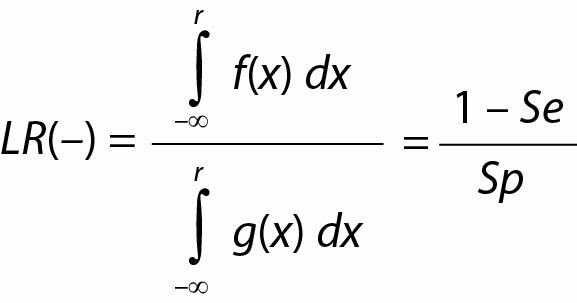


Graphically, *LR*(*–*) is the slope of the line segment joining the cut-off point on the ROC curve to the upper-right corner of the unit square (gray dash dotted line, Figure 2, and Table 1). It is also the area under the curve *f*(*x*) to the left of the cut-off line, line *x* = *r* (false-negative rate = 1 – *Se*, yellow plus the red-hatched area in Figure 1) divided by the area under the curve *g*(*x*) to the left of the cut-off line (true-negative rate = *Sp*, green plus the blue-hatched area in Figure 1).

### Likelihood ratio for a range of test results

Suppose that we want to decrease the cut-off value from *r* to *s* (Figure 1). Graphically, this corresponds to moving along the ROC curve from the solid circle up and to the right to the solid rectangle (Figure 2). Here, we want to examine the likelihood of having a test value between *s* and *r* in those with a disease compared with those without the disease. This is particularly important for tests with polytomous results, say scores obtained from a questionnaire used to categorize people into those with no, mild, moderate, and severe depression. We can define the likelihood ratio for an interval, *LR*(Δ), as follows (*4*, *5*):


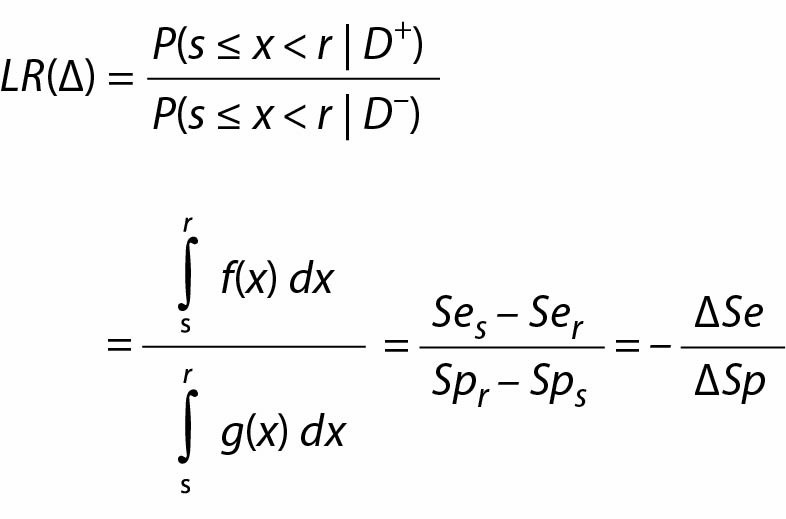


where indices indicate the *Se* and *Sp* for the cut-off values of *r* and *s* (Figures 1 and 2). Graphically, it is equal to the slope of the line segment joining the two points on the ROC curve corresponding to the two cut-off points (grey dash dot dotted line, Figure 2, and Table 1). It also corresponds to the ratio between the red-hatched and blue-hatched areas in Figure 1.

## Example

Suppose the fasting blood sugar (FBS) concentration has a binormal distribution in a group of studied people, having a mean of 89.7 (SD 5.0) mg/dL in healthy people and 99.7 (SD 7.2) in a group of patients with diabetes mellitus. The data presented in Figures 1 and 2 are based on these assumptions. The test values *r* and *s* are 98 and 93 mg/dL, respectively.

As mentioned earlier, *LR*(*r*) for an FBS of 98 mg/dL, is very hard to derive precisely in general. However, assuming the binormal distribution of FBS in our example, then we can easily calculate the density functions for *f*(*r*) and *g*(*r*) using either the MS Excel^®^ function NORMDIST() or *R* function dnorm(). For example, based on the above information, using the Excel function, *f*(*r*) is then NORMDIST(98, 99.7, 7.2, FALSE), which is equal to 0.0539. Using the *R* function, the *g*(*r*) is dnorm(98, mean = 89.7, sd = 5), which is 0.0201. Note that you do not need to use both functions; one is enough. Here, we just used both to show how to use these functions. *LR*(FBS = 98 mg/dL), the slope of the tangent line to the ROC curve corresponding to the point *r*, *f*(*r*) / *g*(*r*), is then 2.68 (= 0.0539 / 0.0201), meaning that an FBS of exactly 98 mg/dL is 2.68 times more likely to be observed in a person with diabetes mellitus as compared with a healthy person.

Now, suppose that the prevalence of diabetes mellitus in the studied population is 0.1. This translates to a pre-test odds of 0.11 [= 0.1 / (1 – 0.1)]. Also, suppose that we take the FBS cut-off value for the diagnosis of diabetes mellitus equal to 98 mg/dL, *i.e.,* those with FBS ≥ 98 mg/dL are considered diabetic. Considering the *Se* of 0.60 and *Sp* of 0.95 (1 – *Sp* = 0.05) at the point on the ROC curve corresponding to *r* (Figure 2), the *LR*(+), the slope of the line segment joining the origin of the unit square to the point corresponding to *r* on the ROC curve, is 12.0 (= 0.60 / 0.05). To determine the post-test odds of the disease, we have:


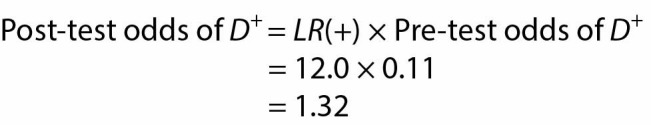


But,


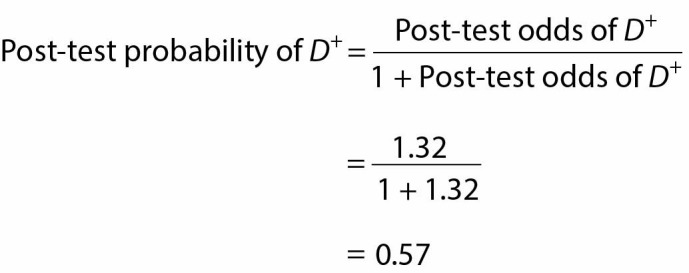


Here, a positive test, having an FBS ≥ 98 mg/dL, increased the probability of diabetes mellitus in a person from 0.1 to 0.57.

Now, what if a person has a negative test result—FBS < 98 mg/dL? Considering the *Se* of 0.60 (1 – *Se* = 0.4) and *Sp* of 0.95 at the cut-off point, *r* (Figure 2), the *LR*(–), the slope of the line joining the point corresponding to *r* on ROC curve to the upper-right corner of the unit square, is 0.42. Then, the post-test odds of having diabetes mellitus is:
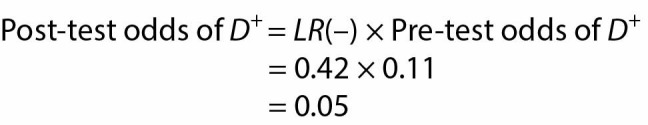
translating to a post-test probability of the disease of 0.05. Notice, when the probability and odds are small, the two values are almost equal.

Finally, to calculate the likelihood ratio of having a FBS between 93 and 98 mg/dL, we need to calculate the slope of the line segment joining the points corresponding to *r* and *s* on the ROC curve (Figure 2). The *Se* and *Sp* of *s* are 0.81 and 0.77. Then we have:
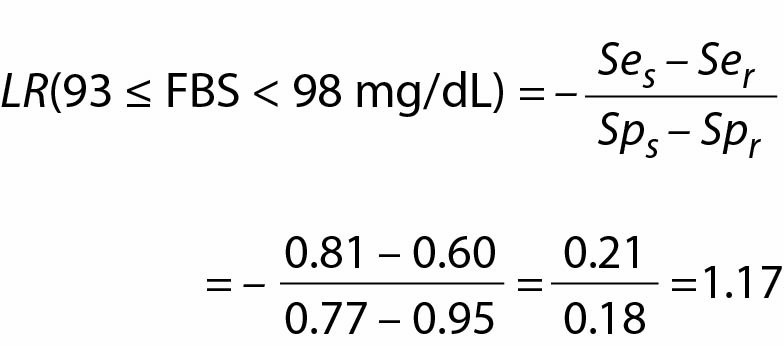
meaning that an FBS between 93 and 98 mg/dL is 1.17 times more likely to be found in a person with diabetes mellitus as compared with a healthy person.

## Conclusion

Having a clear understanding of the meaning and usage of the likelihood ratio is of paramount importance in correct interpretation of test results. Graphical representation of test indices is very helpful in better understanding of this issue. Attention should be paid not to get confused about the likelihood ratio for a specific test result, for a positive or negative test results, and for a range of test values.
